# Association of multidimensional frailty and dynapenia with fall risk in older adults

**DOI:** 10.1186/s12877-025-06097-z

**Published:** 2025-07-02

**Authors:** Maryam Ghorbanzadeh, Afsaneh Bakhtiari, Karimollah Hajian-Tilaki, Maryam Abbaszadeh-Amirdehi

**Affiliations:** 1 Student Research Committee, Babol University of Medical Sciences, Babol, Iran; 2 Social Determinants of Health Research Center, Health Research Institute, Babol university of Medical Sciences, Babol, Iran; 3 Mobility Impairment Research Center, Health Research Institute, Babol University of Medical Sciences, Babol, Iran

**Keywords:** Fall risk, Frailty, Physical frailty, Social frailty, Psychological frailty, Dynapenia, Dynapenic obesity

## Abstract

**Background:**

Dynapenia and frailty are known to be strong predictors of decreased function and increased mortality in the older adults. However, limited research on the co-occurrence of these factors and the different frailty domains makes their relationship with fall risk poorly understood. The aim of this study was to investigate the relationship between fall risk, dynapenia phenotypes, and frailty domains in the older adults.

**Methods:**

In this cross-sectional study of 400 outpatients aged 65+, without major functional, cognitive, or serious comorbid issues, fall risk was assessed using the Berg Balance Scale. Frailty was evaluated via the TFI across physical, psychological, and social domains. Handgrip dynamometry determined dynapenia, classifying the participants into robust, dynapenic, abdominally obese, and dynapenic-obese phenotypes. To examine the associations between frailty, dynapenia, and fall risk, we applied a comprehensive, multi-layered analytical strategy covering all plausible combinations. Associations were analyzed using univariate and multivariate regressions in SPSS v23.

**Results:**

In univariate analyses, fall risk was markedly higher among older adults with dynapenia (59.2%) or multidimensional frailty (60%), and peaked when both conditions coexisted (67.9%). Multivariate models demonstrated that dynapenia (OR = 2.75; 95% CI: 1.48–5.12) and frailty (OR = 2.56; 95% CI: 1.39–4.70) were independently associated with increased fall risk, with a pronounced effect when both were present (OR = 20.45; 95% CI: 6.02–46.69). Among frailty components, only physical (OR = 1.39; 95% CI: 1.19–1.62) and social frailty (OR = 1.38; 95% CI: 1.03–1.84) remained significant predictors in adjusted analyses. Interaction effects revealed that combining dynapenia with these domains further heightened the risk. A clear dose-response pattern emerged: fall risk escalated from 3.24-fold (95% CI: 1.48–3.09) with two frailty domains plus dynapenia, to 7.27-fold (95% CI: 3.46–7.29) when all three domains co-occurred.

**Conclusion:**

Dynapenia and frailty, particularly in physical and social domains, independently and jointly elevate fall risk in older adults. Their co-occurrence demonstrates a dose-dependent effect, underscoring the need for integrated assessments to better identify and manage those at highest risk.

**Supplementary Information:**

The online version contains supplementary material available at 10.1186/s12877-025-06097-z.

## Introduction

Age-related changes in neuromuscular and skeletal factors lead to a decrease in muscle quality, which is which plays a central role in the age-associated reduction of muscle strength. These changes include alterations in muscle fiber morphology, interstitial fat infiltration, motor neuron loss, and impaired neuromuscular transmission [1]. Research consistently shows that low muscle strength is a stronger predictor of functional decline, falls, and mortality than low muscle mass [2]. In 2008, Clark and Manini introduced the the concept of dynapenia, defined as the loss of muscle strength, and/or mechanical power, as a distinct and potentially more functionally important condition than sarcopenia [3].

Dynapenia is prevalent contributor to morbidity in older adults and is strongly linked to decreased physical performance and higher mortality risk. When combined with abdominal obesity, dynapenia has been shown to aggravate metabolic disorders [4, 5], functional limitations [2], falls [1], and mortality [3]. Reported prevalence of dynapenia ranges from 14.1 to 43%, depending on age, sex, diagnostic criteria, and measurement tools [4, 5].

Frailty, another major concern of aging, is a complex, multisystem syndrome that affects the social, psychological, and physical aspects of older adults’ lives. It is associated with chronic diseases, dependence, cognitive and functional decline, lower quality of life, prolonged hospital stays, and increased mortality [6]. While most studies focused on physical frailty using the phenotype models [7], fewer have addressed psychological and social domains [8–10]. Consequently, frailty prevalence varies by assessment method and population. A meta-analysis in 62 countries found a prevalence of 12% based on physical domain, rising to 24% with the frailty index [11]. Although most frailty research comes from Western countries, studies in developing nations, including Iran, are limited. Existing Iranian reports show varied prevalence: one study found 14.3% frail and 25.7% pre-frail [12], while another reported that 46.7% of participants were frail [13].

The link between physical frailty and falls is well established [14], but, associations between social and psychological frailty and fall remain underexplored, despite evidence connecting social isolation [15] and weak social ties [16] with fall risk. Globally, 35–40% of older adults experience falls annually, making it the third leading cause of fatal injuries in the U.S [17]. In Iran, 20–28% of older adults report falls [18], with prevalence ranging from 6.7 to 44% based on age and residence [19]. Nearly half of these falls result in injury [20].

Although the relationships between dynapenic obesity, physical frailty, and falls have been studied individually, the combined impact of frailty domains and dynapenia on fall risk remains largely unexplored. Given that both conditions are potentially reversible, understanding their interplay with fall risk is essential for timely identification and effective prevention. This study aimed to investigate the relationship between fall risk, dynapenia phenotypes (including dynapenic obesity), and frailty domains (physical, psychological, and social) in older adults.

## Methods

### Study design

**Fig. 1:**
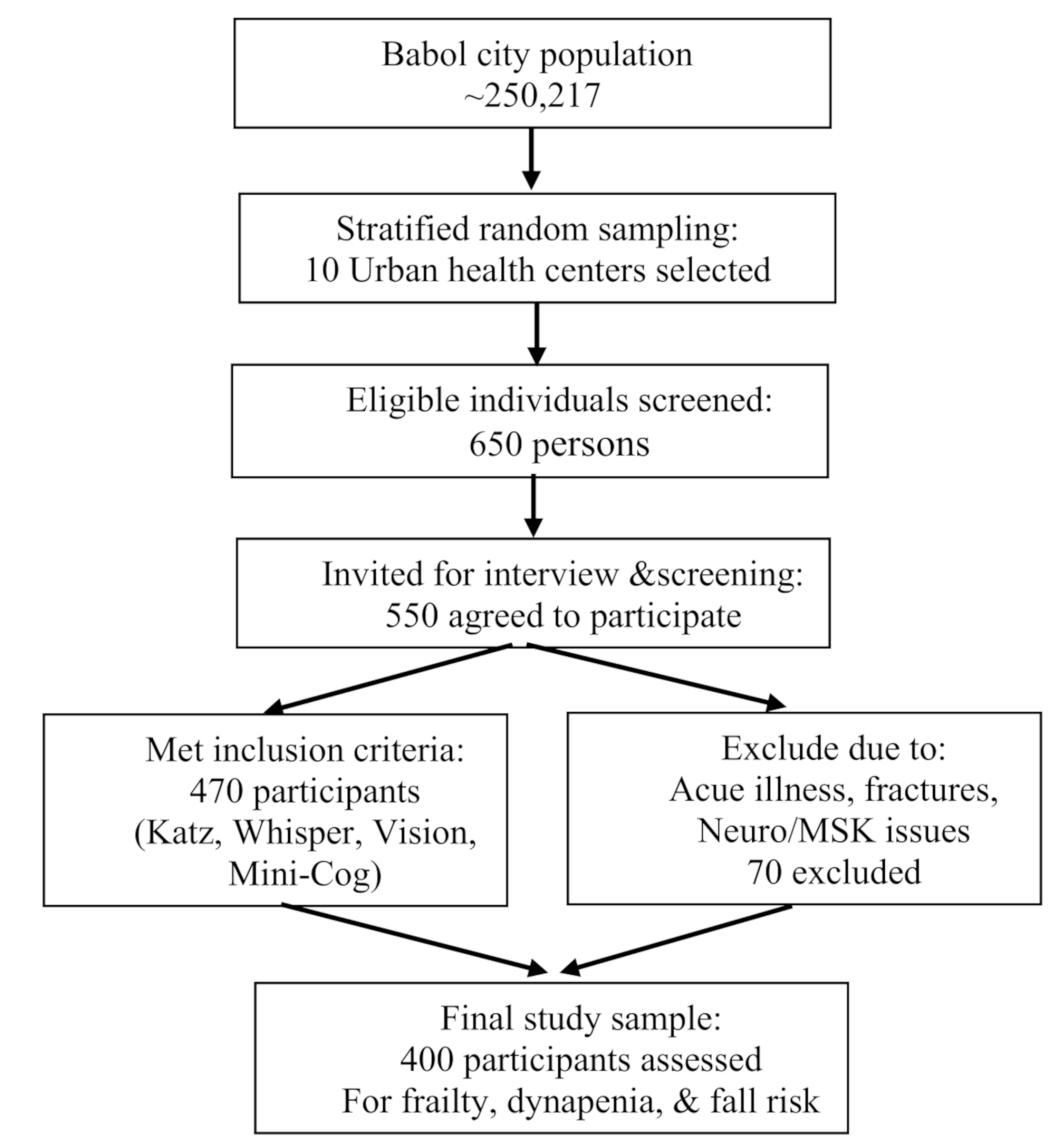
The participant recruitment and selection process in the study

This population-based, cross-sectional study was conducted in Babol City, located in Mazandaran Province, Iran, with a population of approximately 250,217. Stratified random sampling was performed across 13 comprehensive urban health service centers. Babol comprises two municipal districts, each treated as a distinct stratum. Five health centers were randomly selected from each district, resulting in a total of 10 centers. Within each selected center, participants were chosen using a simple random method from eligible clients, based on proportional allocation relative to the district populations. This sampling approach ensured adequate representation and minimized selection bias. The sampling was conducted between August and December 2021 (See Fig. 1).

### Participant’s recruitment and data collection

Participants were randomly selected from the electronic health records of urban health centers. Those identified were contacted and, upon agreement, invited for in-person interviews to complete standardized questionnaires. Written informed consent was obtained beforehand. Inclusion required aged 65 years or older, functional independence and absence of major sensory or cognitive impairments (screened by Katz index, whisper test, WHO vision test, and Mini-Cog respectively). Exclusion criteria included recent acute illness, surgery, fractures, or neurological and musculoskeletal conditions that could interfere with performance-based assessments. These criteria ensured that participants were physically and cognitively able to complete performance-based assessments such as balance and strength tests. The study was approved by the National Ethics Committee of Biomedical Research in Iran (IR.MUBABOL.REC.1400.019) and conducted in accordance with the Declaration of Helsinki and STROBE guidelines.

### Sample size

The estimated sample size for this study was determined as follows:

Given the prevalence of fall risk in individuals without frailty, which is assumed to be 20% according to studies in similar communities [21], and considering a 95% confidence level and 80% test power to detect a difference in the effect of falls (P1-P2 = 0.08) between two groups (those with frailty and those without frailty), a total of 380 individuals were estimated to be needed. Additionally, accounting for a potential 5% dropout rate, the sample size was adjusted to 400 individuals to ensure adequate representation for determining the risk of falling in the study.

### Frailty assessment (independent variable)

Frailty was assessed using the Tilburg Frailty Indicator (TFI), a self-reported instrument designed to capture the multidimensional nature of frailty. This tool was selected due to several advantages it holds over other commonly used screening measures. While many frailty assessments focus primarily on the physical domain or are tailored to clinical settings, the TFI offers a broader scope, evaluating physical, psychological, and social components of frailty [22]. In addition, it has been validated within diverse cultural contexts, including Iran, ensuring its suitability for the current study population [23]. The TFI consists of 15 items distributed across three domains: 8 items related to physical aspects, 4 items addressing psychological concerns, and 3 items focused on social engagement.

Scores range from 0 to 15, with higher values reflecting more severe frailty. A total score of 5 or greater is indicative of frailty. Importantly, the tool also permits analysis of each domain independently. Physical frailty scores range from 0 to 8, psychological from 0 to 4, and social from 0 to 3. To more precisely assess how each domain correlates with fall risk, Receiver Operating Characteristic (ROC) curves were used to determine optimal cut-off values for domain-specific frailty, detailed in Supplementary Table 1.

### Dynapenia assessment (independent variable)

Muscle strength was evaluated using handgrip strength, which served as the principal indicator for identifying dynapenia. Measurements were taken using a digital hand-held dynamometer (Desi Kala Co., Iran), adhering to standardized procedures outlined in the National Health and Nutrition Examination Survey (NHANES) protocol [24]. Participants performed the test in a seated position, with the elbow flexed at 90 degrees, and the highest value recorded from three attempts on the dominant hand was considered. Dynapenia was defined using sex-specific thresholds: <30 kg for men, < 20 kg for women [25].

To identify abdominal obesity, we measured waist circumference (WC) using a flexible tape measure. The measurement was taken after several natural breaths, positioning the tape midway between the lowest palpable rib and the iliac crest along the mid-axillary line, following established anthropometric guidelines [26]. Based on Iranian-specific data, a WC of 95 cm or more was used as the threshold for both sexes [27].

Participants were then stratified into four distinct groups based on the presence or absence of dynapenia and abdominal obesity:

- Robust– neither dynapenia nor abdominal obesity present.
- Non-dynapenic with abdominal obesity.
- Dynapenic without abdominal obesity.
- Dynapenic with abdominal obesity– individuals exhibiting both conditions.

### Fall risk assessment (dependent variable)

We used the Berg Balance test (BBS) to assess fall risk in our participants, a widely recognized tool for community-dwelling older adults [28]. The Berg test, particularly its shortened version, BBS-9, consists of nine tasks rated on a 5-point Likert scale. Reliability of the BBS-9 has been established in Iran [29]. Tasks involve maintaining specific positions, with a maximum score of 36. A cutoff score of 32 indicates fall risk (scores 0–32), while scores equal to or greater than 33 suggest no fall risk [30].

### Personal characteristics

We collected a comprehensive set of personal characteristics through a self-report questionnaire, including age, gender, occupation, educational background, retirement status, economic status adequacy, marital status, living arrangement (lived alone, with a spouse, with married children), polypharmacy (taking at least four medications per day) [31], medical history, including ischemic heart disease, high blood pressure, dyslipidemia, diabetes mellitus, rheumatoid arthritis, osteoporosis, asthma, chronic obstructive pulmonary disease, thyroid disease, kidney failure, and liver cirrhosis, multimorbidity (having at least two chronic diseases) [32], hospitalization history during the previous year, self-perceived health compared to their peers, current smoking status.

### World health organization vision test

To confirm that participants could independently read and comprehend the questionnaire content, their visual abilities were initially evaluated using a widely accepted screening approach recommended by the World Health Organization. This assessment required individuals to identify characters of varying sizes presented at a fixed distance. Only those who demonstrated visual acuity of 6/18 or better in their better-seeing eye, after correction if needed, were included. Those with significant visual limitations who could not read the items unaided were excluded to maintain the integrity of the self-reported data [33].

### The whispered voice test

This test is a simple method for initial screening of hearing impairment in the older adults. In this test, the assessor whispers a few numbers or words from a distance of about 60 cm behind the subject’s head, without the lips being visible. The subject must repeat the phrases heard. Inability to correctly repeat the three suggested words or numbers may indicate mild to moderate hearing loss [34].

### Katz ADL index

The Katz ADL Index is a tool for assessing the functional status of older adults. It assesses six basic self-care activities: bathing, dressing, toileting, transferring, bowel control, and feeding. Each activity is scored as independent (score 1) or dependent (score 0), and the total score ranges from 0 (completely dependent) to 6 (completely independent). A higher score indicates a higher level of functional independence [35].

### Mini-cog

The tool composed of two tasks: three-word recall and the clock drawing test. The procedure begins with presenting the individual with three unrelated words to memorize. Next, the person is asked to draw a clock indicating a specific time, typically 11:10. Finally, they are prompted to recall the original three words. Interpretation is as follows: if no words are recalled, cognitive impairment is likely. If all three words are recalled, the result is considered negative. In cases where one or two words are recalled, the accuracy of the clock drawing determines the outcome—an abnormal clock suggests cognitive impairment [36].

### Statistical analysis

Normality of continuous variables was assessed using the Kolmogorov-Smirnov test. Descriptive statistics were then calculated to provide an overview of the sample characteristics. Univariate associations were tested using chi-square and t-tests. To thoroughly explore the relationships between frailty, dynapenia, and fall risk, a structured multi-step statistical strategy was employed, examining their independent and combined associations. We tested the null hypotheses that there is no association between frailty/dynapenia and fall risk using both unadjusted and adjusted logistic regression models, with adjustments made for potential confounders. The analysis proceeded in multiple stages.

- At first, separate logistic regression models examined the associations of frailty and dynapenia with falls. And then a combined model included both variables to assess their cumulative effect.
- We also analysed each frailty domain (physical, psychological, and social) individually analyzed using logistic regression to examine its association with fall risk. Subsequently, the four-dynapenia phenotypes were assessed in the same manner.
- Further models assessed frailty domains (physical, psychological, and social) separately and in combination with dynapenia to detect domain-specific associations.
- Finally, we analyzed the cumulative burden of frailty by counting the number of frailty domains, with and without dynapenia, to understand how the extent of frailty influences fall risk. Every statistical test was conducted in duplicate, with a significance threshold of *P* < 0.05. IBM SPSS Statistics version 23 (IBM Corp., Chicago, IL, USA) was used for data analysis.

## Results

The participants had a mean age of 72.3 ± 5.9 years (range: 65–89). Table 1 provides a summary of their sociodemographic and clinical characteristics. Statistical analyses revealed significant associations between participants characteristics and the presence of frailty, dynapenia, and fall risk (Supplementary Tables 2–8). Older age, female gender, living alone, multimorbidities, and polypharmacy were linked to higher rates of falls, frailty and its domains, and dynapenia, while higher education was associated with reduced fall risk.

**Table 1:** The individual and clinical participants’ characteristics (*n* = 400)

Variables		Frequency	Percentage
**Gender**	Female	119	29.7
	Man	282	70.3
**Education**	Illiterate	56	16.8
	Elementary	137	41.1
	High school	24	7.2
	≥Diploma	116	38.4
**Receiving pension**	Yes	248	61.8
**Marriage status**	Married	328	82.9
	Single	73	17.1
**Economic adequacy**	Income deficit	176	43.9
	Balanced income	216	53.9
**Live with**	Alone	51	12.7
	Relatives	350	87.2
**Polypharmacy**	Yes	222	57.2
**Multimorbidity**	Yes	236	58.8
**Hospitalization in the past year**	Yes	56	15
**Exersice ≥ 30 min/day**	Yes	227	57.6
**Smoking**	Current smoker	56	15.1
	Former smoker	79	13.4
	Never a smoker	266	71.5
**Perceived health status**	Worse than peers	35	9.5
	Does not matter	150	40.9
	Better than peers	182	49.6
**Fall risk**	Yes	205	51.1
**Dynapenia**	Yes	299	74.6
**Frailty**	Yes	285	71.1
**Frailty + dynapenia**	Yes	215	53.6
**Physical frailty**	Yes	234	58.4
**Pshychological frailty**	Yes	233	58.1
**Social frailty**	Yes	339	84.5
**≥ 2 Frailty dimension**	Yes	178	44.5
**BMI**	< 18.5	6	1.5
	18.5–24.9	153	38.2
	25-29.9	146	36.4
	≥ 30	96	23.9

As shown in Table 2, univariate analyses revealed that fall risk was significantly higher among frail individuals (60%) and those with dynapenia (59.2%) compared to their healthier counterparts (29.3% and 27.5%, respectively). The risk was highest (67.9%) in participants with both conditions, versus approximately 35% in those with either alone. Moreover, 64.4% of individuals with dynapenic obesity were at risk of falling. Mean scores in all three-frailty domains, physical, psychological, and social, were also significantly higher among those at risk (*p* < 0.001).

**Table 2:** The relationship between frailty, dynapenia, and their subgroups with falling risk in older adults

	Fall risk		*p*
	Yes (*n*, %)	No (*n*, %)	
**Frailty**			
Yes	171(60)	114(40)	< 0.001
No	(29.3)34	(70.7)82	
**Frailty domains**			
Physical	159(67.9)	75(32.1)	< 0.001
Psychological	134(57.5)	99(42.5)	< 0.001
Social	187(55.2)	152(44.8)	< 0.001
**Dynapenia**			
Yes	(59.2)177	(40.8)122	< 0.001
No	(27.5)28	(72.5)74	
**Frailty + Dynapenia**			
Only frailty	(35.7)25	(64.3)45	< 0.001
Only dynapenia	(36.9)31	(63.1)53	
Dynapenia + frailty	(67.9)146	(32.1)69	
**Dynapenia Phenotypes**			
Robust	25(31.6)	54(68.4)	< 0.001
Non-dynapenia + abdominal Obesity	3(16.7)	15(83.3)	
Dynapenia - non abdominal Obesity	32(46.4)	37(53.6)	
Dynapenia + abdominal obesity	139(64.4)	77(35.6)	

Univariate associations were tested using chi-square and t-tests

The numbers are frequency and (percentage), or mean ± standard deviation

For testing the null hypothesis, multivariate analyses confirmed that frailty increased fall risk by 3.61 times unadjusted (CI: 2.27–5.75) and 2.56 times after adjustment (CI: 1.39–4.70). This suggests that frailty is a significantly associated with fall risk in older adults, even after adjustment. Similarly, dynapenia increased the odds of falling by 3.83-fold before adjustment and 2.75-fold after adjustment (CI: 2.34–6.27; CI: 1.48–5.12), highlighting the role of muscle weakness in increasing fall risk (Supplementary Tables 9–10). When frailty and dynapenia were considered together, the risk of falling increased even further, emphasizing their combined effect. Specifically, the risk of falling was 5.65-fold (CI: 1.59–20.1) with dynapnea, 5.37-fold with frailty (CI: 1.48–19.41), and 20.45-fold when both were present (CI: 6.02–46.69), with these results remaining significant for moderators (Table 3). The wider confidence intervals likely reflect smaller subgroup sizes and the added variability introduced by adjusting for multiple factors. This made the estimates less precise in some cases.

**Table 3:** Logistic regression to predict the risk of falling based on the simultaneous presence of frailty and dynapenia in the older adults

Frailty–Dynapenia Status	Non-adjusted				Adjusted			
	OR	*p*	95% CI for OR		OR	*p*	95% CI for OR	
			Lower	Upper			Lower	Upper
**Dynapenia**	65.5	007.0	1.59	20.1	56.5	04.0	04.1	68.29
**Frailty**	37.5	0.010	48.1	41.19	1.5	0.05	98.0	26.41
**Dynapenia + Frailty**	45.20	001.0	02.6	46.69	97.12	002.0	65.2	63.38

Adjusted for age, gender, education, living status, polypharmacy and multiomorbidity

Ref: Robust

Unadjusted models indicated that physical, psychological, and social frailty were each associated with greater fall risk (ORs ~ 1.5–1.65, *P* = 0.001). After adjustment, only physical and social frailty remained significant (OR = 1.39, CI: 1.19–1.62; OR = 1.38, CI: 1.03–1.84) (Supplementary Table 11). Table 4 showed that dynapenic obesity posed a greater fall risk than dynapenia alone, up to 11.2 times higher (CI:6.98–12.02), remaining significant after adjustment.

**Table 4:** Association between dynapenia phenotypes and fall risk: logistic regression analysis

Dynapenia Phenotypes	Non-adjusted				Adjusted			
	OR	*p*	95% CI for OR		OR	*p*	95% CI for OR	
			Lower	Upper			Lower	Upper
Non-dynapenia + Abdominal obesity	2.77	0.15	0.70	2.98	1.51	0.35	0.50	3.26
Dynapenia- Non Abdominal obesity	5.63	0.01	1.41	6.43	4.41	0.02	1.40	6.96
Dynapenia + Abdominal obesity	11.18	0.001	6.98	12.02	9.89	0.001	7.07	10.12

Adjusted for age, gender, education, living status, polypharmacy and multiomorbidity

Ref: Robust

To examine domain-specific associations, each frailty domain was paired with dynapenia (Supplementary Table 12). All combinations increased fall risk in unadjusted models; post-adjustment, only physical and social frailty retained significance. In a comprehensive model including all three frailty domains and dynapenia, only physical frailty (OR = 1.59, CI: 1.36–1.86) and dynapenia (OR = 88.3, CI: 2.26–6.64) were independently associated with higher fall risk (Table 5).

**Table 5:** Combined effects of dynapenia and frailty domains on fall risk: unadjusted and adjusted logistic regression models

Frailty domains + dynapenia	Unadjusted				Adjusted			
	OR	*P*	95% CI for OR		OR	*P*	95% CI for OR	
			Lower	Upper			Lower	Upper
**Physical**	1.59	0.001	1.36	1.86	1.43	0.001	1.19	1.72
**Psychological**	1.03	0.79	0.80	1.32	0.83	0.28	0.60	1.16
**Social**	1.08	0.58	0.81	1.43	1.15	0.43	0.80	1.67
**Dynapenia**	3.88	0.001	2.26	6.64	2.93	0.001	1.53	5.58

Adjusted for age, gender, education, living status, polypharmacy and multiomorbidity

Ref: Robust

Finally, cumulative frailty burden analysis (Table 6) showed a graded increase in fall risk with more coexisting frailty domains alongside dynapenia, from 3.24 times higher with two domains (CI: 1.48–3.09) to 7.27 times with all three (CI: 3.46–7.29), even after adjustment..

**Table 6:** Logistic regression to predict the risk of falling based on the number of presence of frailty domains with dynapenia in the elderly

Frailty domains count + dynapenia	Non-adjusted				Adjusted			
	OR	*p*	95% CI for OR		OR	*p*	95% CI for OR	
			Lower	Upper			Lower	Upper
**One domain**	1.82	0.15	0.80	4.18	1.11	0.83	0.41	3.04
**Two domains**	3.24	0.003	1.48	3.09	2.38	0.04	1.97	5.83
**Three domains**	7.27	0.001	3.46	7.29	3.63	0.003	1.54	4.54
**Dynapenia**	4.21	0.001	2.50	5.09	3.91	0.001	2.13	4.21

Adjusted for age, gender, education, living status, polypharmacy and multiomorbidity

Ref: Robust

## Discussion

This study aimed to assess the relationship between frailty, dynapenia, and fall risk. The findings showed that both frailty and dynapenia independently increased fall risk, with a 5.65-fold increase for dynapenia, 5.37-fold for frailty, and 20.45-fold when both were present. These associations remained significant after adjusting for confounders such as age, gender, education, living status, polypharmacy, and multimorbidities. Moreover, fall risk was higher when at least two frailty domains coexisted with dynapenia. After adjustment, the coexistence of physical and social frailty with dynapenia showed the strongest association with fall risk, supporting the value of assessing multiple frailty domains, while psychological frailty did not show a significant association. Although the association between physical frailty and fall risk is well-established [37, 38], limited research exists on the role of social and psychological frailty, particularly in relation to dynapenia, in fall risk. Our study aims to addresses this gap in the literature.

Regarding social factors, previous studies have similarly indicated a significant association with fall risk, in line with our findings, emphasizing their critical role in elderly vulnerability to falls [39, 40]. Pohl et al. conducted a longitudinal study showing the link between social isolation and falls [15]. Another study revealed that individuals with poor and moderate social connections had a hazard ratio of 1.7 and 1.5, respectively, for traumatic falls compared to those with strong social ties. Furthermore, functional decline was twice as high among those with poor social support and severe/multiple falls compared to those with good social connections [16]. Social frailty increases fall risk through mechanisms such as isolation, leading to reduced physical activity, limited access to support, and increased vulnerability, which negatively influence mobility and balance. Although the concept of social frailty in relation to falls has received limited attention, it refers to the ongoing risk of losing essential social resources needed throughout life. Social frailty should not only address the absence of social resources but also the lack of social behaviors, activities, and self-management abilities [8]. It accelerates physical and mental decline, with social roles potentially diminishing before physical and cognitive functions [9].

The concept of psychological frailty, including cognitive, emotional, and motivational domains, has also received limited attention in geriatric research. It reflects to subtle brain-related changes that go beyond normal aging, without reaching the threshold of clinical disease, potentially reducing resilience to emotional or cognitive stress and increasing vulnerability to adverse outcomes over time [41]. Older adults experiencing psychological challenges such as depression and anxiety may show lower physical activity levels, indirectly raising their risk of falls. Prior studies, including those by Luo et al. [42] and Davoodi et al. [43], have reported significant links between these psychological factors and increased fall risk due to diminished motivation and activity. However, in our study, psychological frailty did not independently affect fall risk after adjustment. This discrepancy may stem from methodological differences. While previous studies targeted specific conditions like depression, we used a validated, multidimensional tool that provided a more comprehensive measure of psychological frailty. This broader approach may have diluted the direct association with falls. Moreover, differences in contextual factors such as supportive living environments, might contribute to varying effects, with those in more supportive settings possibly experiencing fewer negative impacts of psychological frailty.

Studies have demonstrated that dynapenic obesity is associated with a higher risk of falls [44, 45]. In our study, the likelihood of falling risk was twice as high among those with dynapenic obesity compared to individuals with dynapenia alone, consistent with findings by Veronese et al. [4]. A 14-year prospective study in an older Chinese population showed that participants with dynapenic abdominal and non-abdominal obesity, as well as those with abdominal obesity without dynapenia, had slower gait speeds than those without either condition. Dynapenic obesity, in both forms, predicted a greater fall risk, highlighting the importance of preserving mobility through muscle strengthening and fat reduction [46]. These findings reinforce the broader evidence that dynapenia, with or without obesity, contributes to adverse health outcomes in older adults. Reduced muscle strength impairs flexibility, balance, and stability, increasing the risk of falls. Age-related changes, such as diminished motor cortex excitability and increased fat infiltration in muscles, further weaken muscle function. Additionally, adipose tissue-derived cytokines may directly suppress muscle force production [1–3].

Emerging evidence suggests a link between dynapenic abdominal obesity and frailty. Our study revealed that when frailty and dynapenia coexist, the risk of falls in older adults rises markedly, beyond the effect of each condition on its own. Notably, this risk was further elevated when dynapenia was accompanied by at least two frailty components, whether physical, psychological, or social. These findings advocate for a multidimensional perspective in evaluating fall risks. As far as we are aware, this is the first investigation to consider the full spectrum of frailty domains, alongside dynapenia, in relation to fall outcomes. In support of our results, Sun et al. [47] found that abdominal obesity, dynapenia, and their combination accelerated the trajectory toward frailty in Chinese older adults, with dynapenia (with or without central fat) having the strongest impact.

Mechanistically, this interplay may reflect shared underlying disturbances: frailty typically involves reduced physiological reserve and impaired balance, while dynapenia contributes to loss of muscular strength and function. Together, they intensify functional instability. Furthermore, factors such as systemic inflammation, insulin resistance, and oxidative stress, common to both conditions, create a metabolic environment that promotes fat infiltration into muscle. This process compromises muscle quality and hastens decline, potentially creating a cycle of muscular and functional deterioration. Ultimately, this constellation of impairments makes individuals significantly more vulnerable to falls. These findings underline the necessity of integrated clinical strategies that simultaneously address dynapenia and the multiple domains of frailty. The holistic relationship among frailty domains, as demonstrated in longitudinal research among European older adults [48], underscores the interconnected nature of frailty domains. These findings highlight the importance of comprehensive assessment of all frailty domains in both clinical practice and research to guide tailored interventions.

Several considerations should be noted when interpreting these findings. First, while all statistical analyses followed pre-specified hypotheses, the multiple comparisons performed may affect result precision. The BBS, though validated, serves as an indirect measure of fall risk rather than capturing actual fall occurrences. The cross-sectional nature restricts causal interpretation. Although associations were adjusted for multiple confounders, temporal relationships cannot be established. Additionally, the male-predominant sample likely reflects cultural patterns in healthcare utilization rather than true population distribution. Recruiting exclusively from clinical settings may impact generalizability. While polypharmacy was adjusted for, drug-specific effects (e.g., sedatives on balance) weren’t examined. Chronic disease severity wasn’t documented, and excluding recent surgical/neuromuscular cases– while methodologically sound– may limit relevance for frailer populations. Future studies could address these through broader sampling and prospective designs. A key strength of this study is its focus on the combined impact of dynapenia and multidimensional frailty, moving beyond the physical aspect alone. By including psychological and social factors, it offers a more complete view of fall risk in older adults. Its clinical relevance is enhanced by using a widely accepted functional tool, and the discussion connects findings to real biological mechanisms, not just numbers.

## Conclusion

The results showed that although frailty and dynapenia are each associated with the falling risk, their simultaneous presence increases the chance of falling by four times. On the other hand, the presence of frailty domains simultaneously with dynapenia increases the chance of falling risk. Therefore, it is suggested to include frailty and dynapenia assessment in health promotion programs for the older adult in primary health care settings. It is also recommended that the assessment of frailty include all its domains and not only the physical domain.

## Electronic supplementary material

Below is the link to the electronic supplementary material.

Supplementary Material 1

## Data Availability

The data that support the findings of this study are available on reasonable request from the corresponding author, [A.B.].

## Abbreviations

TFI: Tilburg frailty indicator
ROC: Receiver operating characteristic
NHANES: The national health and nutrition examination survey
WC: Waist circumference
BBS: Berg balance test
