# Supplementary material for: Association of multidimensional frailty and dynapenia with fall risk in older adults
Source: BMC Geriatr. 2025 Jul 2;25:442. doi: 10.1186/s12877-025-06097-z (PMC12219132; doi:10.1186/s12877-025-06097-z)
Supplement: Supplementary file 1 — Supplementary Material 1 [file 12877_2025_6097_MOESM1_ESM.docx]

**Supplementary Table – Method**

Table 1 Cutoff points determined for frailty dimensions based on ROC curve.

| Min-Max score | **Cutoff points** | **Specificity** | **Sensitivity** | **Frailty dimensions** |
| --- | --- | --- | --- | --- |
| 0-8 | 3 | 1 | 0.72 | Physical |
| 0-4 | 2 | 1 | 0.73 | Psychology |
| 0-3 | 1 | 0.93 | 0.85 | Social |

**Supplementary Table – Results**

**Table 2** The relationship between age with the risk of frailty, falls, and dynapenia in elderly

| **p-value** | **SD ± Mean** | **Variables** | |
| --- | --- | --- | --- |
| 0.001 | 50.6± 34.74 | Yes | **Risk of falling** |
|  | 29.4± 23.70 | No |  |
| 0.001 | 10.6±15.73 | Yes | **Dynapenia** |
|  | 48.4±96.69 | No |  |
| 0.202 | 08.6±57.72 | Yes | **Total frailty** |
|  | 40.5±75.71 | No |  |
| 0.003 | 18.6±08.73 | Yes | **Physical frailty** |
|  | 31.5±29.71 | No |  |
| 0.719 | 96.5±42.72 | Yes | **Psychological frailty** |
|  | 82.5±21.72 | No |  |
| 0.815 | 87.5±36.72 | Yes | **Social frailty** |
|  | 10.6±17.72 | No |  |

As age increased, there was a notable and significant rise in the risk of falling, dynapenia, and physical frailty.

**Table 3** Relationship of gender with the risk of frailty, falls, and dynapenia in elderly

| **Frailty +**  **Dynapenia** | | **Social Frailty** | | **Psychological Frailty** | | **Physical Frailty** | | **Frailty** | | **Dynapenia** | | **Falling**  **Risk** | | **Variables** |
| --- | --- | --- | --- | --- | --- | --- | --- | --- | --- | --- | --- | --- | --- | --- |
| **No** | **Yes** | **No** | **Yes** | **No** | **Yes** | **No** | **Yes** | **No** | **Yes** | **No** | **Yes** | **No** | **Yes** |  |
| 42  (35.3) | 77^a^  (64.7) | 7  (5.9) | 112^a^  (94.1) | 35  (29.4) | 84^a^  (70.6) | 33  (27.7) | 86^a^  (72.3) | 19  (16.0) | 100^a^  (84.0) | 28  (23.5) | 91  (76.5) | 33  (27.7) | 86 ^a^  (72.3) | **Female** |
| 144  (51.0) | 138  (48.9) | 55  (19.5) | 227  (80.5) | 133  (47.2) | 149  (52.8) | 134  (47.5) | 148  (52.5) | 97  (34.4) | 185  (65.6) | 74  (26.2) | 208  (73.8) | 163  (57.8) | 119  (42.2) | **Male** |

Numbers represent frequency (percentage).

^a^. p<0.001

^b^. p<0. 01

^c^. p<0.05

The risk of falls, frailty and its dimensions, as well as dynapenia + frailty, were significantly higher in women compared to men.

**Table 4** The relationship between education with the risk of frailty, fall risk, and dynapenia in elderly

| **Frailty +**  **Dynapenia** | | **Social Frailty** | | **Psychological Frailty** | | **Physical Frailty** | | **Frailty** | | **Dynapenia** | | **Falling**  **Risk** | | **Variables** |
| --- | --- | --- | --- | --- | --- | --- | --- | --- | --- | --- | --- | --- | --- | --- |
| **No** | **Yes** | **No** | **Yes** | **No** | **Yes** | **No** | **No** | **Yes** | **No** | **Yes** | **No** | **Yes** | **No** |  |
| 23  (41.07) | 33  (58.9) | 6  (10.7) | 50  (89.3) | 16  (28.6) | 40  (71.4) | 18  (32.1) | 38  (67.9) | 9  (16.1) | 47  (83.9) | 22  (39.3) | 34 ^a^  (60.7) | 24  (42.9) | 32^b^  (57.1) | Illiterate |
| 55  (40.14) | 82  (59.9) | 22  (16.1) | 115  (83.9) | 47  (34.3) | 90  (65.7) | 49  (35.8) | 88  (64.2) | 34  (24.8) | 103  (75.2) | 28  (20.4) | 109  (79.6) | 57  (41.6) | 80  (58.4) | Elementary |
| 10  (41.66) | 14  (58.3) | 4  (16.7) | 20  (83.3) | 8  (33.3) | 16  (66.7) | 8  (33.3) | 16  (66.7) | 7  (29.2) | 17  (70.8) | 6  (25.0) | 18  (75.0) | 10  (41.7) | 14  (58.3) | High school |
| 70  (60.34) | 46  (39.7) | 21  (18.1) | 95  (81.9) | 64  (55.2) | 52  (44.8) | 46  (55.2) | 52  (44.8) | 42  (36.2) | 74  (63.8) | 37  (31.9) | 79  (68.1) | 84  (72.4) | 32  (27.6) | ≥Diploma |

Numbers represent frequency (percentage).

^a^. p<0.001

^b^. p<0. 01

^c^. p<0.05

The risk of falling decreased with increasing education.

**Table 5** The relationship between married status with the risk of frailty, falls, and dynapenia in elderly

| **Frailty+**  **Dynapenia** | | **Social Frailty** | | **Psychological Frailty** | | **Physical Frailty** | | **Frailty** | | **Dynapenia** | | **Falling**  **Risk** | | **Variables** | |
| --- | --- | --- | --- | --- | --- | --- | --- | --- | --- | --- | --- | --- | --- | --- | --- |
| **No** | **Yes** | **No** | **Yes** | **No** | **Yes** | **No** | **No** | **Yes** | **No** | **Yes** | **No** | **Yes** | **No** |  |  |
| 161  (50.31) | 159^a^  (49.7) | 57  (17.8) | 263^a^  (82.2) | 145  (45.3) | 175^b^  (54.7) | 144  (45.0) | 176^c^  (55.0) | 105  (32.8) | 215^a^  (67.2) | 85  (26.6) | 235  (73.4) | 171  (53.4) | 149^a^  (46.6) | **Married** |  |
| 17  (25.8) | 49  (74.2) | 3  (4.5) | 63  (95.5) | 19  (28.8) | 47  (71.2) | 19  (28.8) | 47  (71.2) | 7  (10.6) | 59  (89.4) | 11  (16.7) | 55  (83.3) | 19  (28.8) | 47  (71.2) | **Single** |  |

Numbers represent frequency (percentage).

^a^. p<0.001

^b^. p<0. 01

^c^. p<0.05

Married individuals showed a significantly lower risk of falls, frailty, dynapenia + frailty, and all three dimensions of frailty compared to those who were single.

**Table 6** The relationship between coexistence with the risk of frailty, falls, and dynapenia in elderly

| **Frailty+**  **Dynapenia** | | **Social Frailty** | | **Psychological Frailty** | | **Physical Frailty** | | **Frailty** | | **Dynapenia** | | **Falling**  **Risk** | | **Variables** | |
| --- | --- | --- | --- | --- | --- | --- | --- | --- | --- | --- | --- | --- | --- | --- | --- |
| **No** | **Yes** | **No** | **Yes** | **No** | **Yes** | **No** | **No** | **Yes** | **No** | **Yes** | **No** | **Yes** | **No** |  |  |
| 16  (31.4) | 35^a^  (68.6) | 3  (5.9) | 48  (94.1) | 13  (25.5) | 38^c^  (74.5) | 15  (29.4) | 36  (70.6) | 6  (11.8) | 45^a^  (88.2) | 11  (21.6) | 40  (78.4) | 15  (29.4) | 36^a^  (70.6) | **Alone** | **Coexistence**  **Status** |
| 159  948.3) | 170  (51.7) | 55  (16.7) | 274  (83.3) | 142  (43.2) | 187  (56.8) | 142  (43.2) | 187  (56.8) | 102  (31.0) | 227  (69.0) | 84  (25.5) | 245  (74.5) | 171  (52.0) | 158  (48.0) | **With relatives** |  |

Numbers represent frequency (percentage).

^a^. p<0.001

^b^. p<0. 01

^c^. p<0.05

Individuals living alone demonstrated a higher risk of falls, psychological frailty, and the combined condition of dynapenia + frailty compared to those who did not live alone.

**Table 7** The relationship between polypharmacy with the risk of frailty, falls, and dynapenia in elderly

| **Frailty+**  **Dynapenia** | | **Social Frailty** | | **Psychological Frailty** | | **Physical Frailty** | | **Frailty** | | **Dynapenia** | | **Falling**  **Risk** | | **Variables** | |
| --- | --- | --- | --- | --- | --- | --- | --- | --- | --- | --- | --- | --- | --- | --- | --- |
| **No** | **Yes** | **No** | **Yes** | **No** | **Yes** | **No** | **No** | **Yes** | **No** | **Yes** | **No** | **Yes** | **No** |  |  |
| 49  (26.48) | 136^b^  (73.51) | 19  (14.8) | 109  (85.2) | 48  (37.5) | 80  (62.5) | 39  (30.5) | 89^a^  (69.5) | 30  (23.4) | 98  (76.6) | 22  (17.2) | 106^b^  (82.8) | 50  (39.1) | 78^a^  (60.9) | **Yes** | **Polypharmacy** |
| 137  (63.42) | 79  (36.57) | 43  (15.8) | 230  (84.2) | 120  (44.0) | 153  (56.0) | 128  (46.9) | 145  (53.1) | 86  (31.5) | 187  (68.5) | 80  (29.3) | 193  (70.7) | 146  (53.5) | 127  (46.5) | **No** |  |

Numbers represent frequency (percentage).

^a^. p<0.001

^b^. p<0. 01

^c^. p<0.05

Individuals experiencing polypharmacy exhibited a significantly elevated risk of falls, dynapenia, physical frailty, and the combined condition of dynapenia + frailty.

**Table 8** The relationship between comorbidity with the risk of frailty, falls, and dynapenia in elderly

| **Frailty+**  **Dynapenia** | | **Social Frailty** | | **Psychological Frailty** | | **Physical Frailty** | | **Frailty** | | **Dynapenia** | | **Falling**  **Risk** | | **Frailty+**  **Variables** | |
| --- | --- | --- | --- | --- | --- | --- | --- | --- | --- | --- | --- | --- | --- | --- | --- |
| **No** | **Yes** | **No** | **Yes** | **No** | **Yes** | **No** | **No** | **Yes** | **No** | **Yes** | **No** | **Yes** | **No** |  |  |
| 131  (43.37) | 171  (56.6) | 41  (13.6) | 261  (86.4) | 118  (39.1) | 184^c^  (60.9) | 114  (37.7) | 188^a^  (62.3) | 77  (25.5) | 225^a^  (74.5) | 75  (24.8) | 227  (75.2) | 133  (44.0) | 169^a^  (56.0) | **Yes** | **Multimorbidity** |
| 55  (55.55) | 44  (44.4) | 21  (21.2) | 78  (78.8) | 50  (50.5) | 49  (49.5) | 53  (53.5) | 46  (46.59) | 39  (39.4) | 60  (60.6) | 27  (27.3) | 72  (72.7) | 63  (63.6) | 36  (36.4) | **No** |  |

Numbers represent frequency (percentage).

^a^. p<0.001

^b^. p<0. 01

^c^. p<0.05

It was observed that individuals with multimorbidity conditions exhibited a significantly higher risk of falling, as well as increased levels of frailty, particularly in the realms of physical and psychological frailty.

**Table 9** Logistic regression for falling risk prediction in elderly: unadjusted and adjusted models based on frailty in elderly

|  | **Unadjusted** | | | | | **Adjusted** | | | | |
| --- | --- | --- | --- | --- | --- | --- | --- | --- | --- | --- |
|  | **B** | **OR** | **p-value** | **95% CI for OR** | | **B** | **p-value** | **OR** | **95% C.I.for OR** | |
|  |  |  |  | **Lower** | **Upper** |  |  |  | **Lower** | **Upper** |
| **Frailty** | 1.28 | 3.61 | 0.001 | 2.27 | 5.75 | 0.94 | 0..001 | 2.56 | 1.39 | 4.70 |

1. Adjusted for age, gender, education, living situation, polypharmacy and multimorbidity.

The table above displays the results of the logistic regression test, investigating the association between frailty and the risk of falling in two scenarios: Non-adjusted and adjusted. The results indicated that both before and after adjustment, frailty significantly increases the likelihood of falling by 3.61 times and 2.56 times, respectively (P=0.001).

**Table 10** Logistic regression for falling risk prediction in elderly: unadjusted and adjusted models based on dynapenia in elderly

|  | **Unadjusted** | | | | | **Adjusted** | | | | |
| --- | --- | --- | --- | --- | --- | --- | --- | --- | --- | --- |
|  | **B** | **OR** | **p-value** | **95% CI for OR** | | **B** | **p-value** | **OR** | **95% CI for OR** | |
|  |  |  |  | **Lower** | **Upper** |  |  |  | **Lower** | **Upper** |
| **Dynapenia** | 1.34 | 3.83 | 0.001 | 2.34 | 6.27 | 1.01 | 0.001 | 2.75 | 1.48 | 5.12 |

1. Adjusted for age, gender, education, living situation, polypharmacy and multimorbidity.

Logistic regression showed that dynapenia significantly increased the risk of falling by 3.83 times before adjustment and 2.75 times after adjustment (P = 0.001).

**Table 11** Logistic regression for falling risk prediction in elderly: unadjusted & adjusted models on the association of frailty domains separately

| **Frailty dimensions** | **Unadjusted** | | | | | **Adjusted** | | | | |
| --- | --- | --- | --- | --- | --- | --- | --- | --- | --- | --- |
|  | **B** | **OR** | **p-value** | **95% CI for OR** | | **B** | **p-value** | **OR** | **95% CI for OR** | |
|  |  |  |  | **Lower** | **Upper** |  |  |  | **Lower** | **Upper** |
| **Physical** | 0.5 | 1.65 | 0.001 | 1.44 | 1.87 | 0.33 | 0.001 | 1.39 | 1.19 | 1.62 |
| **Psychological** | 0.42 | 1.53 | 0.001 | 1.27 | 1.84 | 0.16 | 0.19 | 1.17 | 0.91 | 1.51 |
| **Social** | 0.44 | 1.55 | 0.001 | 1.25 | 1.93 | 0.32 | 0.026 | 1.38 | 1.03 | 1.84 |

1. Adjusted for age, gender, education, living situation, polypharmacy and multimorbidity.

The results showed that in the unadjusted state, physical frailty increased the risk of falls by 1.65 times, psychological frailty by 1.53 times, and social frailty by 1.55 times (P=0.001). In the adjusted state, only physical frailty (P=0.001) and social frailty (P=0.026) remained significant.

**Table 12** Logistic regression for falling risk prediction in elderly: unadjusted & adjusted models on the association of frailty–dynapenia pairing with dynapenia

| **Frailty–dynapenia pairing** | **Unadjusted** | | | | | **Adjusted** | | | | |
| --- | --- | --- | --- | --- | --- | --- | --- | --- | --- | --- |
|  | **B** | **OR** | **p-value** | **95% CI for OR** | | **B** | **p-value** | **OR** | **95% CI for OR** | |
|  |  |  |  | **Lower** | **Upper** |  |  |  | **Lower** | **Upper** |
| **Physical** | 0.49 | 1.64 | 0.001 | 1.43 | 1.87 | 0.34 | 0.001 | 1.41 | 1.2 | 1.64 |
| **Dynapenia** | 1.34 | 3.38 | 0.001 | 2.24 | 6.55 | 1.07 | 0.001 | 2.93 | 1.54 | 5.59 |
| **Psychological** | 0.44 | 1.55 | 0.001 | 1.28 | 1.89 | 0.18 | 0.16 | 1.19 | 0.92 | 1.54 |
| **Dynapenia** | 1.37 | 3.96 | 0.001 | 2.93 | 6.57 | 1.03 | 0.001 | 2.8 | 1.5 | 5.23 |
| **Social** | 0.49 | 1.63 | 0.001 | 1.3 | 2.05 | 0.35 | 0.019 | 1.42 | 1.06 | 1.91 |
| **Dynapenia** | 1.42 | 4.15 | 0.001 | 2.5 | 6.9 | 1.05 | 0.001 | 2.87 | 1.53 | 5.38 |

1. Adjusted for age, gender, education, living situation, polypharmacy and multimorbidity.

Logistic regression analysis for falling risk prediction based on the association of dynapenia with each frailty dimension separately showed that, in the unadjusted model, physical, psychological, and social frailty, when associated with dynapenia, significantly increased the risk of falling. After adjustment, only physical frailty (P = 0.001) and social frailty (P = 0.019) remained statistically significant.
